# The Potential Roles of Post-Translational Modifications of PPARγ in Treating Diabetes

**DOI:** 10.3390/biom12121832

**Published:** 2022-12-08

**Authors:** Xiaohui Ji, Wenqian Zhang, Liqin Yin, Zunhan Shi, Jinwen Luan, Linshan Chen, Longhua Liu

**Affiliations:** School of Exercise and Health, Shanghai University of Sport, Shanghai 200433, China

**Keywords:** post-translational modifications, PPARγ, diabetes, thiazolidinediones

## Abstract

The number of patients with type 2 diabetes mellitus (T2DM), which is mainly characterized by insulin resistance and insulin secretion deficiency, has been soaring in recent years. Accompanied by many other metabolic syndromes, such as cardiovascular diseases, T2DM represents a big challenge to public health and economic development. Peroxisome proliferator-activated receptor γ (PPARγ), a ligand-activated nuclear receptor that is critical in regulating glucose and lipid metabolism, has been developed as a powerful drug target for T2DM, such as thiazolidinediones (TZDs). Despite thiazolidinediones (TZDs), a class of PPARγ agonists, having been proven to be potent insulin sensitizers, their use is restricted in the treatment of diabetes for their adverse effects. Post-translational modifications (PTMs) have shed light on the selective activation of PPARγ, which shows great potential to circumvent TZDs’ side effects while maintaining insulin sensitization. In this review, we will focus on the potential effects of PTMs of PPARγ on treating T2DM in terms of phosphorylation, acetylation, ubiquitination, SUMOylation, O-GlcNAcylation, and S-nitrosylation. A better understanding of PTMs of PPARγ will help to design a new generation of safer compounds targeting PPARγ to treat type 2 diabetes.

## 1. Introduction

By 2021, there were over 537 million people with diabetes in the world, and in China, the number had exceeded 140 million [1,2]. Diabetes (diabetes mellitus) is a metabolic disorder caused by defective insulin secretion or insulin resistance, or both, resulting in chronic hyperglycemia (elevated plasma glucose levels) with glucose, lipid, and protein metabolic dysfunctions [3]. Type 2 diabetes mellitus (T2DM) accounts for over 90% of cases of diabetes and is caused by compensatory secretion deficiency or insulin resistance, usually accompanied by microvascular (retinopathy, nephropathy, neuropathy) and macrovascular (stroke, myocardial infarction, amputation) complications [4]. Diabetes causes tremendous psychological and physical suffering to patients and places a huge burden on the health care system. The tremendous economic cost of diabetes is conservatively projected to make up 2.2% of the global GDP by 2030 [5].

Treatment of T2DM requires alleviation of insulin resistance and correction of dyslipidemia while preserving pancreatic β-cell function [4]. Peroxisome proliferator-activated receptor γ (PPARγ) is a ligand-activated nuclear receptor, the activation of which can heighten the sensitivity of peripheral tissues to insulin and therefore increase glucose uptake and utilization in muscle, promote adipocyte remodeling, fatty acid decomposition, and synthesis in adipose tissue while inhibiting gluconeogenesis in liver [6,7]. Thiazolidinediones (TZDs), PPARγ agonists, are crucial to modulating glucose and lipid metabolic homeostasis [8,9] and mainly act by increasing insulin sensitivity [10]. Moreover, it has been found in clinical studies that pioglitazone has potential therapeutic efficacy in reducing the risk of cerebrovascular disease in T2DM patients [9]. However, the use of TZDs has been prohibited in several countries due to their detriments, such as heart failure, weight gain, and edema [11]. Ergo, it has become an unprecedented challenge to develop a new drug targeting PPARγ that could maintain TZD’s insulin-sensitizing effects while alleviating its adverse effects.

PPARγ expression and transcriptional activity can be regulated by post-translational modifications (PTMs) [12], including phosphorylation, SUMOylation, ubiquitination, GlcNAcylation, and acetylation [13]. These PTMs differently modulate PPARγ activity in adipogenesis and insulin sensitivity, through which there is potential to treat T2DM with fewer side effects than TZDs [14]. In this review, we elaborate on the various PTMs of PPARγ and further summarize the biological effects of different modifications on PPARγ from the perspectives of modification sites, amino acid sequences, and related research progress. The exploration of whether different PTMs can partially activate PPARγ to sharpen insulin sensitivity with fewer side effects may provide novel insight into the drug design for treating T2DM.

## 2. Domain Structure of PPARγ

The three different family members of PPARs (PPARα, PPARβ/δ, and PPARγ) play different roles in glucose and lipid metabolism. Besides its anti-inflammatory function, PPARα is also crucial in the transport of lipoprotein [7]. PPARβ/δ could stimulate AMP-activated protein kinase (AMPK) to improve glucose metabolism, fatty acid oxidation, endoplasmic reticulum stress, and inflammation [15].

The human PPARγ gene is located at position 3p25 on chromosome 3, within the 1.5 trillion enzymes (Mb) of D3S1263, a suitable polymorphism marker for lipid metabolism-related diseases [16]. In mice, the PPARγ gene is located at position E3-F1 on chromosome 6 [17]. Both human and mouse PPARγ genes have more than 100 kb of genomic DNA and generate two isoforms, PPARγ1 and PPARγ2. The PPARγ1 gene is encoded by eight exons, including two γ1-specific exons for the untranslated region at the 5′-end, A1 and A2, as well as six other exons coding identical sequences for these two isoforms. PPARγ2 is encoded by seven exons, with the first exon B encoding additional *N*-terminal amino acids specifically for PPARγ2 [18,19,20,21,22].

**Figure 1:**
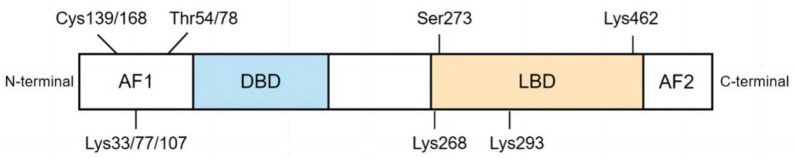
Structural characteristics and species conservation of PPARγ. Schematic diagram of the PPARγ protein structure includes the activation function 1 and 2 domains (AF1 and AF2), DNA binding domain (DBD), and ligand-binding domain (LBD).

From the perspective of molecular structure, the activity of PPARγ is mediated by different ligands, with respective effects on its structural conformation and dynamic properties. Compared with PPARγ1, PPARγ2 contains 30 more amino acids in the *N*-terminal activation domain and is mainly expressed in adipose tissue, while PPARγ1 is more widely expressed [23,24]. Insulin can enhance the activity of the PPARγ *N*-terminal domain and promote its transcriptional activity [24]. The activation function 1 domain (AF1) of PPARγ is a highly flexible one that cooperates with regulatory proteins, which binds to Lys33, Lys37, Lys107, Thr54, Thr78, Cys139, and Cys168 residues of PPARγ (Figure 1) [25]. The AF2 domain is an activating functional region located at the C terminus. DNA binding domain (DBD) is a functional region of transcriptional activator proteins that can identify and bind DNA. The LBD domain is the major region in which PPAR is modified, such as Lys268, Ser273, Lys293, and Lys462.

## 3. PPARγ Agonists and T2DM

PPARγ synthetic agonists include members of the thiazolidinedione (TZD) class, such as the total agonists rosiglitazone and pioglitazone, which are widely applied in the treatment of T2DM due to their capability to enhance the sensitivity of target tissues to insulin and improve glucose metabolism. Among the clinical antidiabetic drugs, thiazolidinediones (TZDs) do not cause hypoglycemia like insulin or insulin secretagogues (i.e., sulfonylureas) and can be used in combination with other antidiabetic drugs to significantly ameliorate insulin sensitivity and provide long-lasting glycemic control [11]. TZDs boost the adipogenesis of peripheral adipocytes, mitigate the activity of visceral adipocytes, and thus reduces triglycerides in the liver and periphery, which can explain the increase in subcutaneous fat after TZDs therapy instead of visceral fat. Furthermore, TZDs can promote adiponectin secretion and reduce plasma free fatty acids, elevate plasma high-density lipoprotein cholesterol, and transform high-density small LDL particles into buoyant LDL particles, which significantly alleviates insulin resistance and metabolic syndrome, denting the demand for insulin [26]. TZDs also exhibit vasodilatory effect, lower systolic and diastolic blood pressure, decrease the production of adhesion molecules, as well as inhibit the proliferation of vascular smooth muscle cells and intimal tissue hyperplasia after coronary artery stenting in T2D patients. PPARγ knockout mice, without any form of adipose tissue, had a complex metabolic phenotype, such as increased lean body mass and organ dysfunction, and developed severe T2D afterward [27]. PPARγ was also involved in the self-renewal of adipocytes since mouse mature adipocytes could only survive for only a few days after selective ablation of PPARγ [28,29,30]. In addition, PPARγ is essential to controlling the network of genes involved in glucose homeostasis, including increased expression of glucose transporter type 4 (GLUT4) and C-CBL-associated proteins (CAP) [31]. Fully activated PPARγ by TZDs such as rosiglitazone could also lead to some adverse effects such as edema, weight gain, and heart failure [8,32].

## 4. Post-Translational Modifications (PTMs) of PPARγ in Diabetes

**Figure 2:**
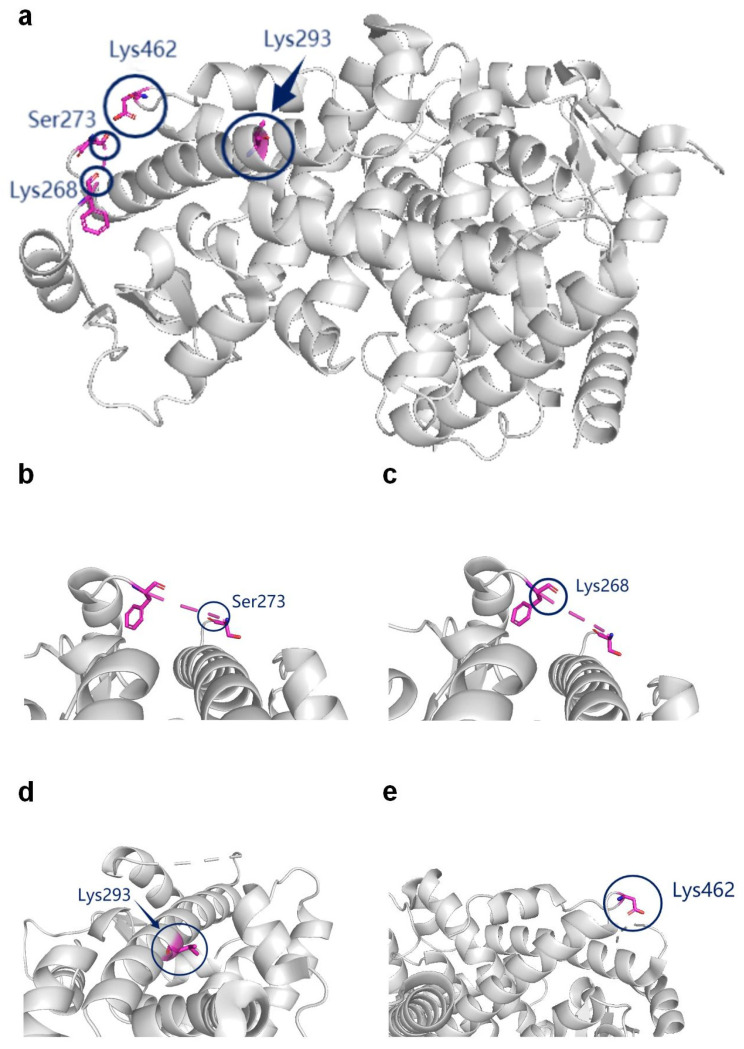
Specific distribution of partial modification sites in the LBD region of PPARγ. (**a**) Modification sites in the LBD region of PPARγ. This structure is a ligand-free structure of human PPARγ LBD. (PDB DOI: 10.2210/pdb6L8B/pdb. NDB: 6L8B) (Accessed on 16 September 2020. www.rcsb.org). (**b**) Schematic diagram of experimental procedures for the detection of Phosphorylated PPARγ. The modification sites for PPARγ phosphorylation are shown in the circle: Ser273. In addition to the site Ser273, the other site where phosphorylation of PPARγ occurs is Thr78, which is located within AF1. (**c**,**d**) Schematic diagram of experimental procedures for the detection of Deacetylated PPARγ. The modification sites for PPARγ phosphorylation are shown in the circle: Lys268, Lys293. (**e**) Schematic diagram of experimental procedures for the detection of Ubiquitinated PPARγ. The modification sites for PPARγ ubiquitination are shown in the circle: Lys462.

PPARγ activity and its critical role in modulating adipocyte development can be emphasized by several covalent modifications [33]. PTMs of PPARγ can increase the functional diversity of this protein, such as protein spatial conformational changes, protein–protein interactions, or affinity regulation between receptors and ligands, and then affect the transcription of PPARγ downstream target genes [34]. Diverse PTMs of PPARγ have been studied, including phosphorylation, ubiquitination, SUMOylation, acetylation and deacetylation, O-GlcNAcylation, and S-nitrosylation. Here, this review will focus on the potential role of PTMs in regulating the activity of PPARγ from the perspective of molecular structure. Several modification sites are marked on the ligand-binding domain (LBD) of PPARγ (6L8B) (Figure 2a). A table of the six modification sites of PPARγ mentioned in this review and their function in diabetes are listed at the end of this section(Table 1).

### 4.1. Phosphorylation

PPARγ can be phosphorylated, and its transcriptional activity, which can be ligand-dependent or independent, is impacted by kinases and phosphatases, such as AMP kinase (AMPK), mitogen-activated protein kinases (ERK- and P38-MAPK) [65].

Phosphorylation can be achieved by mitogen-activated protein kinases (MAPK) [66], including extracellular signal-regulated kinase, p38, and c-Jun amino-terminal kinase [67]. MAPK can inhibit adipogenesis by mediating PPARγ phosphorylation [68]. Mediated by the cyclin-dependent kinase 5 (CDK5), phosphorylation of PPARγ can significantly reduce insulin sensitivity and induce obesity [35]. At the active site of CDK5, Ser273 of PPARγ (Figure 2b) is regulated by the transient unfolding of β-strand (β1) on the surface near the consensus region. Rosiglitazone and Pioglitazone, two representatives of TZDs, can effectively protect PPARγ from phosphorylation at Ser273, inhibiting the activity of Cdk5, which leads to increased fatty acid oxidation and the expression of mitochondrial proteins [35,36]. It has been widely acknowledged that reversing the obesity-related phosphorylation of PPARγ at Serine 273 (Ser273) improved insulin sensitivity [35,37]. Genetically modified mice encoding an allele of PPARγ cannot be phosphorylated at Ser273 and could protect from insulin resistance probably by inhibiting Gdf3 [37]. Meanwhile, direct stabilization of the hydrophobic region between H3 and β1–β4 blocks the phosphorylation of PPARγ [69]. Inhibitory Ser112 phosphorylation of PPARγ in white adipose tissue is mediated by PDGFRβ signaling, and the PDGFR antagonist imatinib can promote adipocyte proliferation and glucose tolerance [38]. UNIST HYUNDAI Compound 1 (UHC1), a new PPARγ ligand non-agonist, blocked CDK5-mediated phosphorylation of PPARγ stronger than rosiglitazone. It does not block CDK5-mediated phosphorylation of Rb but inhibits the TNF-α-mediated phosphorylation of PPARγ in adipocytes, suggesting that PPARγ could be selectively directed and significantly improved insulin sensitivity in high-fat-fed mice without causing significant side effects, which means it could be a potential therapeutic agent for T2D and related metabolic diseases [39]. It is also worth noting that c-Src kinase was shown to directly phosphorylate PPARγ at Tyr78, and inhibition of it may aggravate insulin resistance and proinflammatory genes in adipose tissue [70]. Hence, phosphorylation of PPARγ at different sites regulates its activity in different pathways and needs further investigation to decipher these regulatory mechanisms.

### 4.2. Acetylation

Acetylation of PPARγ also regulates its own activity. Histone acetyltransferases include p300, CREB-binding protein (CBP), and four classes of HDACs. The four classes of HDACs are similar in DNA sequence and function [71], including: class I: HDAC1 to HDAC3, HDAC8; class II: HDAC4 to HDAC7, HDAC9, HDAC10; class III: SIRT1 to SIRT7; and class IV: HDAC11. Acetylation can occur on histones and non-histones and can significantly affect their function [72]. Histone H3 acetylation in the promoter region of PPARγ regulates expression during adipogenesis, enabling the differentiation of preadipocytes into adipocytes, and the degree of fat accumulation is closely related to the degree of acetylation [73]. Studies of primary white adipose tissue cells and preadipocyte 3T3-L1 cell lines from mice showed that rosiglitazone could reduce the acetylation of PPARγ [73].

In contrast to acetylation, deacetylase SirT1, which triggers ligand-dependent regulation of PPARγ deacetylation, may be a regulatory switch for the conversion of white adipose tissue to brown adipose tissue [41]. Deacetylation of PPARγ at K268 and K293 (Figure 2c,d) can set off a series of reactions, ranging from recruiting brown adipose tissue program coactivators, promoting white fat browning to increasing thermogenesis, exhibiting increased energy expenditure, significantly suppressing obesity and improving insulin sensitivity (same as TZDs), to reducing fat accumulation, bone loss, edema, and congestive heart failure [42]. The study of mice with deacetylation-mimetic PPARγ mutations K268R/K293R proved that PPARγ deacetylation can selectively regulate target genes, inhibit lipid oxidation genes with an anti-atherosclerotic effect in the treatment of diabetes, and the improvement of endothelial function [43]. Furthermore, PPARγ deacetylation prevents mice from aging-related atherosclerosis with anti-inflammatory function [46].

Thus, PPARγ deacetylation appears to be able to highlight its metabolic advantages by promoting brown fat remodeling decoupling side effects, making it possible to design safer and more efficient PPARγ agonists to treat diabetes.

### 4.3. Ubiquitination

FBXO9, an E3 ligase, is an important enzyme for regulating PPARγ through ubiquitination, which can affect PPARγ stability and activity [46]. Ubiquitination, controlled by enzymes E1, E2, and E3, can sequentially activate, bind, and link ubiquitin to substrate proteins (mainly lysine residues), causing covalent linkage of ubiquitin. It also controls eukaryotic activities by promoting protein ubiquitination and degradation [74]. Ubiquitin E3 ligase mediates the transfer of ubiquitin from the E2 ubiquitin-binding enzyme to specific substrate proteins [75]. PPARγ is an E3 ubiquitin ligase, and its ligand domain can bind to multiple protein structures and induce the ubiquitination and degradation of selenin S (SelS) and SelK to promote adipocyte differentiation. Data from preadipocyte 3T3-L1 overexpression and knockout SelS or SelK experiments suggest that PPARγ-mediated ubiquitination and degradation of SelS and SelK are essential for adipocyte differentiation [76]. Ubiquitination degradation of NFκb/p65 by Lys48-linked polyubiquitin, which is delivered by ligand-binding domains, inhibits the proinflammatory response [77]. The key role of Lys462 (Figure 2e) in ubiquitination on PPARγ has been demonstrated by site-directed mutagenesis [47]. Adipocyte-specific knock-down of CUL4B-RING E3 ligase (CRL4B), which promotes polyubiquitination and proteasomal degradation of PPARγ, can ameliorate high fat-induced glucose intolerance and insulin resistance, as well as enhance adipocyte insulin sensitivity [78].

Overexpression of ubiquitin ligase F-box only protein 9 (FBXO9) and MKRN1 induces ubiquitination of PPARγ, significantly reduces endogenous PPARγ activity, and inhibits adipogenesis [46,48]. Neural progenitor cells express developmentally downregulated protein 4 (NEDD4), an E3 ubiquitin ligase that interacts with the hinge and ligand-binding domains of PPARγ. PPARγ expression is positively correlated with NEDD4 expression in obese adipose tissue. Knockout of NEDD4 in preadipocyte 3T3-L1 can decrease PPARγ protein levels [49]. Muscle-specific ubiquitin ligase muscle ring finger-2 (MuRF2), a ubiquitin ligase that regulates cardiac PPARα and PPARγ1 activity, aggravates high-fat diet-induced diabetic cardiomyopathy in mice [50].

Therefore, de-ubiquitination regulates the stability and activity of endogenous and exogenous PPARγ [47,51], increases glucose and fatty acids intake, decreases blood glucose and triglyceride levels, and reduces lipid accumulation and degeneration in the organs, which seems to be a very effective target for antidiabetic drug development.

### 4.4. SUMOylation

The SUMOylating of a protein is a reversible process, a cascade catalyzed by a series of enzymes, including the activating (E1), conjugating (E2), and ligating (E3) enzymes. It can be reversed by a series of sumo-specific proteases (SENPs) [79]. In the E3 ligase, PIAS1 plays the most critical role [52]. The PIAS family includes PIAS1, PIAS3, PIASxα, and PIASxβ, among which PIAS1 is a rate-limiting enzyme [53]. Similar to ubiquitination, SUMOylation enhances protein–protein interactions by conjugation of peptide bonds to lysine residues in target proteins in three sequential steps, including changes in cellular localization, protein activity, or protein stability. Post-translational SUMOylation and de-SUMOylation modifications are relatively mild compared to the complexity of the ubiquitination/de-ubiquitination machinery [80]. Studies suggest that SUMOylation of PPARγ occurs only within the *N*-terminal activating function 1 (AF1) domain (mainly at lysine 33 and lysine 77) and inhibits both basal and ligand-induced activation of PPARγ1. Ligands that bind to the C-terminal ligand-binding domain (LBD) of PPARγ1 can only reduce SUMOylation at lys33 [81]. Blocking the SUMOylation of PPARγ at Lys107 in mice can achieve enhanced insulin sensitivity without causing excessive obesity [82].

Excessive SUMOylation of PPARγ induces insulin resistance and vascular endothelial dysfunction along with an endogenous cascade that intensifies that reaction [54]. SUMO-specific protease 1 (SENP1) unblocks PPARγ transcription and adipocyte differentiation, enhancing adipogenesis. In contrast, adipocyte SENP2 knockout mice developed insulin resistance and ectopic fat accumulation, resulting in reduced fat storage [55,56]. Fibroblast growth factor 21 (FGF21) knockout (KO) mice exhibit defective PPARγ signaling, attenuated gene expression, impaired insulin secretion, plasma non-esterified fatty acid and triglyceride concentrations, and increased hepatic triglycerides concentration. However, rosiglitazone treatment in FGF21 KO mice was ineffective, which was consistent with this significant increase in PPARγ SUMOylation, and replenishment of FGF21 reversed PPARγ SUMOylation and restored activity [57]. It also showed that carbon monoxide could inhibit lipopolysaccharide (LPS) proinflammatory responses in macrophages by enhancing SUMOylation of PPARγ through mitochondrial reactive oxygen species (ROS) production [58]. Ligand-dependent SUMOylation of the LBD of PPARγ, an inflammatory gene promoter, prevents the recruitment of corresponding LPS signals and inhibits the inflammatory response of macrophages [83].

The evidence above may suggest further exploration of the insulin signaling pathway regulated by PPARγ SUMOylation, and inhibition of PPARγ SUMOylation is a potential way to design novel anti-T2D vascular complications drugs.

### 4.5. O-GlcNAcylation

O-GlcNAc is dynamically regulated by two highly conserved enzymes, namely, the O-GlcNAc transferase (OGT)and the O-GlcNAcase (OGA). The OGT transfers the GlcNAc from the UDP-GlcNAc to either an amino threonine residue or to a specific target protein filament [72]. O-GlcNAcylation adds the single monosaccharide β-O-d-*N*-acetylglucosamine (β-O-GlcNAc) to serine or threonine residues in the nucleus, cytoplasm, mitochondrial, and almost every functional class of proteins [84]. O-GlcNAc modification acts as a nutritional sensor and regulator of insulin signaling pathways. Elevated levels of O-GlcNAc stimulate glucose uptake, which is directly related to insulin resistance and hyperglycemic-induced glucose toxicity, and may be associated with the development of diabetes and diabetic complications [85,86]. The primary modification site is threonine 54 in the *N*-terminal AF-1 domain of PPARγ, where its modification can reduce lipid accumulation and surrounding phospholipid-1 expression, and its specific O-GlcNAcase inhibitor can inhibit 30% PPARγ transcriptional activity and terminal adipocyte differentiation in preadipocyte 3T3-L1 [60]. The underlying mechanisms of O-GlcNAcylation and metabolism- and senescence-related chronic diseases, such as diabetes, cancer, and neurodegeneration, have been reviewed from many perspectives [87]. However, the few studies on its association with the T2D target PPARγ seem to be a promising research direction.

### 4.6. S-Nitrosylation

S-nitrosylation refers to the modification that part of the nitroso group (nitric oxide group) is covalently linked to the sulfhydryl residue of a specific cysteine residue in the protein to form S-nitroso, resulting in S-nitroso protein [88]. GSNOR is a highly evolutionarily conserved S-nitrosylated glutathione (GSNO) reductase that regulates protein sulfhydryl nitrosylation by metabolizing GSNO [62]. In diabetic mice, proinflammatory macrophages nitrosylate PPARγ in adipocytes via inducible nitric oxide synthase. S-nitrosylation of PPARγ results in impaired function, including inhibiting its transcriptional activity, reducing protein stability and adiponectin expression, which disrupts the balance of maintaining insulin sensitivity [63]. The S-nitrosylated denitrotinase modification of PPARγ also alters the balance between adipogenic and osteogenic differentiation in bone marrow mesenchymal stem cells, and the absence of this enzyme shows increased S-nitrogenation of PPARγ, decreased binding to downstream target fatty acid-binding protein 4 (FABP4), exhibits fat loss, and a wasting phenotype in mice [64]. The present study identified Cys 139 and Cys168 of PPARγ as S-nitrosylation sites. This may suggest that S-nitrosylation of PPARγ may be a “bad” modification in diabetes treatment, increases adipocyte production, and impairs insulin sensitivity.

## 5. Discussion and Conclusions

With a deeper understanding of T2DM and its related clinical practice, precision medicine in diabetes get more and more recognized in recent years, which purpose is to develop personalized and progressive programs for patients with diabetes [89,90]. In mechanistic studies of diabetes, as a well-established drug target for diabetes, PPARγ has been studied for decades. Full activation of PPARγ through TZDs could improve insulin sensitivity but with several adverse effects that impeded these compounds from wide use. Selectively activated PPARγ demonstrates the potential of maintaining insulin sensitivity with less adverse effects. Post-translational modification of PPARγ provides us with a great opportunity to fulfill this goal.

In this review, in terms of molecular structure, the activity of PPARγ can be regulated by different ligands, which shows the specific distribution of some modification sites through the LBD region of PPARγ. Modification changes at different sites also affect the activity of the PPARγ gene and its key role in regulating glucose and lipid metabolism. PTMs can increase the functional diversity of the protein. Here, we comprehensively summarized six well-studied PTMs of PPARγ and proposed that inhibition of PPARγ SUMOylation, phosphorylation, and acetylation, appears to be ways to un-couple the beneficial metabolic effects of antidiabetic drugs from their adverse effects in drug design and development. De-phosphorylation, de-SUMOylation, de-ubiquitination, deacetylation, as well as stabilization of O-GlcNAcylation, and reduction in S-nitrosylation may be novel ways of treating T2D. However, there are few studies that focus on the interaction between these post-translational modifications. Whether inhibition of one modification has a “superposition” or “antagonism” effect on other modificational sites is still unknown. How to carry out more refined modification and regulation to achieve the optimal safe “anti-T2D” effect remains to be further investigated. In the near future, more and more selective agonists of PPARγ based on its PTMs may help with treating T2DM with less adverse effects.

## Figures and Tables

**Table 1:** PPARγ modification site and function in diabetes.

Modification Type	Site	Enzyme	Domain	Function in Diabetes	Refs.
Phosphorylation	Ser273, Ser112, Tyr78,	MAPK	N-A/B	↓insulin sensitivity, obesity ↑insulin resistance and proinflammatory genes in adipose tissue	[35,36,37,38,39,40]
Acetylation	Lys268, Lys293	p300, CBP, HDACs	C-LBD	↓insulin sensitivity, energy expenditure, differentiation of preadipocytes into adipocytes ↑fat accumulation, bone loss, edema, and congestive heart failure	[41,42,43,44,45]
Ubiquitination	Lys107, Lys462	FBXO9	N-AF1, C-LBD	↓glucose tolerance, adipocyte insulin sensitivity, stability of endogenous and exogenous PPARγ, ↑insulin resistance, blood glucose and triglyceride levels, glucose and fatty acid uptake diabetic cardiomyopathy	[46,47,48,49,50,51]
SUMOylation	Lys107, Lys33, Lys77	E1, E2, E3	N-AF1, C-LBD	↑insulin resistance, lipogenesis, obese, adipose tissue accumulation, inflammation, vascular endothelial dysfunction,	[52,53,54,55,56,57,58,59]
O-GlcNAcylation	Thr54	OGT, OGA	N-AF1	↑PPARγ transcriptional activity, adipocyte differentiation, hyperglycemia-induced transcriptional activation of multiple genes	[60,61]
S-nitrosylation	Cys168, Cys139	GSNOR	N-AF1	↓adiponectin expression, transcriptional activity, protein stability, insulin sensitivity, adipogenic differentiation of BMSCs ↑adipocyte generation	[62,63,64]

## Data Availability

Not applicable.
